# Differential Effect of Extracellular Vesicles Derived from *Plasmodium falciparum*-Infected Red Blood Cells on Monocyte Polarization

**DOI:** 10.3390/ijms24032631

**Published:** 2023-01-30

**Authors:** Ladawan Khowawisetsut, Sinmanus Vimonpatranon, Kittima Lekmanee, Hathai Sawasdipokin, Narinee Srimark, Kesinee Chotivanich, Kovit Pattanapanyasat

**Affiliations:** 1 Department of Parasitology, Faculty of Medicine Siriraj Hospital, Mahidol University, Bangkok 10700, Thailand; 2 Siriraj Center of Research Excellence for Microparticle and Exosome in Diseases, Research Department, Faculty of Medicine Siriraj Hospital, Mahidol University, Bangkok 10700, Thailand; 3 Graduate Program in Immunology, Department of Immunology, Faculty of Medicine Siriraj Hospital, Mahidol University, Bangkok 10700, Thailand; 4 Department of Clinical Tropical Medicine, Faculty of Tropical Medicine, Mahidol University, Bangkok 10400, Thailand

**Keywords:** *Plasmodium falciparum*, extracellular vesicles, monocytes, polarization, malaria

## Abstract

Malaria is a life-threatening tropical arthropod-borne disease caused by *Plasmodium* spp. Monocytes are the primary immune cells to eliminate malaria-infected red blood cells. Thus, the monocyte’s functions are one of the crucial factors in controlling parasite growth. It is reasoned that the activation or modulation of monocyte function by parasite products might dictate the rate of disease progression. Extracellular vesicles (EVs), microvesicles, and exosomes, released from infected red blood cells, mediate intercellular communication and control the recipient cell function. This study aimed to investigate the physical characteristics of EVs derived from culture-adapted *P. falciparum* isolates (*Pf*-EVs) from different clinical malaria outcomes and their impact on monocyte polarization. The results showed that all *P. falciparum* strains released similar amounts of EVs with some variation in size characteristics. The effect of *Pf*-EV stimulation on M1/M2 monocyte polarization revealed a more pronounced effect on CD14^+^CD16^+^ intermediate monocytes than the CD14^+^CD16^−^ classical monocytes with a marked induction of *Pf*-EVs from a severe malaria strain. However, no difference in the levels of microRNAs (miR), miR-451a, miR-486, and miR-92a among *Pf*-EVs derived from virulent and nonvirulent strains was found, suggesting that miR in *Pf*-EVs might not be a significant factor in driving M2-like monocyte polarization. Future studies on other biomolecules in *Pf*-EVs derived from the *P. falciparum* strain with high virulence that induce M2-like polarization are therefore recommended.

## 1. Introduction

Malaria is an acute febrile illness caused by an apicomplexan protozoan, *Plasmodium* spp. It is one of the serious infectious diseases that cause human deaths in tropical and subtropical countries. Among human-infected *Plasmodium* spp., *P. falciparum* (*Pf*) is a crucial species, because it is widely distributed in malaria endemic countries, has a high infection prevalence, and results in severe diseases, often with fatal outcomes [1].

Humans acquire the infection mostly from mosquito bites that release sporozoites to the blood circulation and infect hepatocytes. After the infected hepatocytes are ruptured, the hepatic merozoites infect red blood cells (RBCs) and begin the erythrocytic life cycle. Several host immune mechanisms work in concert to eliminate the parasites and parasite-infected cells and control the infection. CD8 T cells are the major cells that destroy the infected hepatocytes [2,3]. The *Plasmodium*-specific antibodies and monocytes/macrophages are the essential host immune mechanisms that serve to control the *Plasmodium* spp. during the intraerythrocytic cycle. These mechanisms block merozoite invasion and phagocytose the infected red blood cells (iRBCs) and free merozoites [4,5]. As monocytes are a type of cell that play a crucial role in eliminating malaria-iRBCs in the circulating blood, the immunoregulation of monocyte functions by protozoa and their antigenic products is interesting and important to explore.

Human monocytes have been subdivided into three populations. These include the classical monocytes (CD14^+^CD16^−^), intermediate monocytes (CD14^+^CD16^+^), and the nonclassical monocytes (CD14^dim^CD16^+^) [6]. Acute malaria infection expands the intermediate and nonclassical monocyte subsets and increases the levels of proinflammatory and regulatory cytokine production [7,8,9]. In addition, these monocytes phagocytose antibody-opsonize malaria-infected erythrocytes more efficiently than other monocyte subsets [10]. The decreased percentages of nonclassical monocytes, which have a role in resolving malaria, have been associated with severity and death in *P. falciparum* malaria patients [11]. Thus, the alteration of monocyte subsets seems to affect malaria pathogenesis and the rate of disease progression.

In addition, the monocytes can be identified based on their function. Thus, M1-like and M2-like monocytes mirror the well-known types of M1/M2 macrophages. The differentiation of M1-like monocytes and M1 macrophages (M1 cells) is mediated throughout the NF-κB pathway, Janus kinases (JAK)-signal transducer and activator of transcription 1 (STAT1) and STAT5 transcription factor pathways. These cells express CD80 on the cell surface and produce iNOS and proinflammatory cytokines. The M2-like monocytes and M2 macrophages (M2 cells) are differentiated through the activation of STAT3, STAT6, and PPAR-γ transcription factors. These cells express CD206 and produce anti-inflammatory cytokines [12,13,14]. In addition, several microRNAs (miRNAs and miR) are involved in macrophage differentiation, such as miR-155, miR-9, miR-127, miR-125b, and let-7a/f, which induce M1 differentiation, whereas miR-146, miR-223, and let-7c/e inhibit M1 cell differentiation. miR-124, miR-223, miR-34a, miR-132, miR-146a, miR-125a-5p, miR-142-5p, and let-7c were involved in M2 cell differentiation [15,16].

Several studies on the M1/M2 monocyte polarization in malaria clinical samples found that the phenotypes of monocytes appear to be related to the severity of the disease. For example, the M2-like blood monocyte phenotype in malaria infection is significantly associated with disease severity in children with falciparum malaria [17]. Furthermore, the monocytes of malaria-infected children with severe complications express high levels of CD11b, CD11c, CD18, HLA-DR, CD86, TNF-α, and interleukin (IL)-6, whereas they express low levels of CD11a, TLR2, and TLR4 compared with those of normal healthy individuals [18].

The monocytes/macrophage can recognize the parasite products via pattern recognition receptors on the cell surfaces, activating the transcription factors and inducing chemokine and cytokine production [19,20,21,22]. The in vitro studies also supported the fact that the malarial products can modulate the monocyte polarization and function. As the extracellular vesicles (EVs) have become new mediators involved in malaria pathogenesis, the increase in EV numbers has been shown to be associated with disease severity [23,24]. Two types of EVs that widely study the function of intercellular communication are microvesicles (MV) and exosomes (Exo). The MV originate from outward budding and fission of the cell membrane. In contrast, Exo are derived from the endosomal compartment, formed as a multivesicular body (MVB), and fused with the cell membrane for release into the extracellular space [25]. The monocytes can uptake EVs secreted by *P. falciparum*-infected red blood cells (*Pf*-EVs), resulting in monocyte activation [21,26,27]. A recent study showed that the internalization of *Pf*-EVs is associated with the sialylated complex N-glycans carried on *Pf*-EVs [28]. Although *Pf*-EVs were reported to have a role in monocyte activation, their effects on monocyte differentiation remain to be studied. Thus, the present study is aimed at exploring the effect of *Pf*-EVs on phenotypic changes of monocytes and their mediation of monocyte polarization. We isolated and characterized the *Pf*-EVs from *P. falciparum* strains with different pathogenicity and studied their effects on primary monocyte polarization.

## 2. Results

### 2.1. Characteristics of EVs Derived from Different P. falciparum Strains

**Figure 1:**
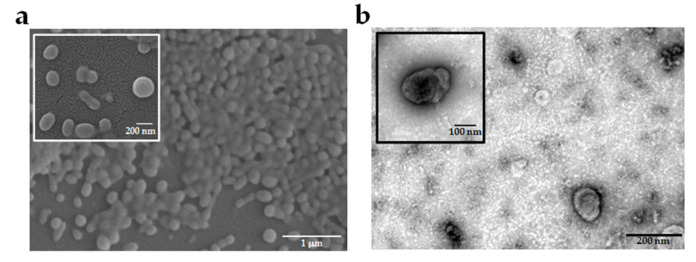
Representative electron micrograph of *Pf*-EVs isolated from a malaria culture supernatant. (**a**) *Pf-*MV imaged by FE-SEM at a magnification of 10,000× with a bar scale 1 μm and 30,000× with a bar scale 200 nm (inset). (**b**) *Pf*-Exo imaged by TEM at a magnification of 15,000× with a bar scale 200 nm and 20,000× with a bar scale 100 nm (inset).

The *Pf*-EVs were isolated from a malaria culture supernatant with multi-step centrifugation. The representative electron micrograph revealed the morphology of *Pf*-EVs. The EVs were spherical-shaped particles when detected by field emission scanning electron microscopy (FE-SEM) and cup-shaped membrane-enclosed particles when detected by transmission electron microscopy (TEM) (Figure 1).

**Figure 2:**
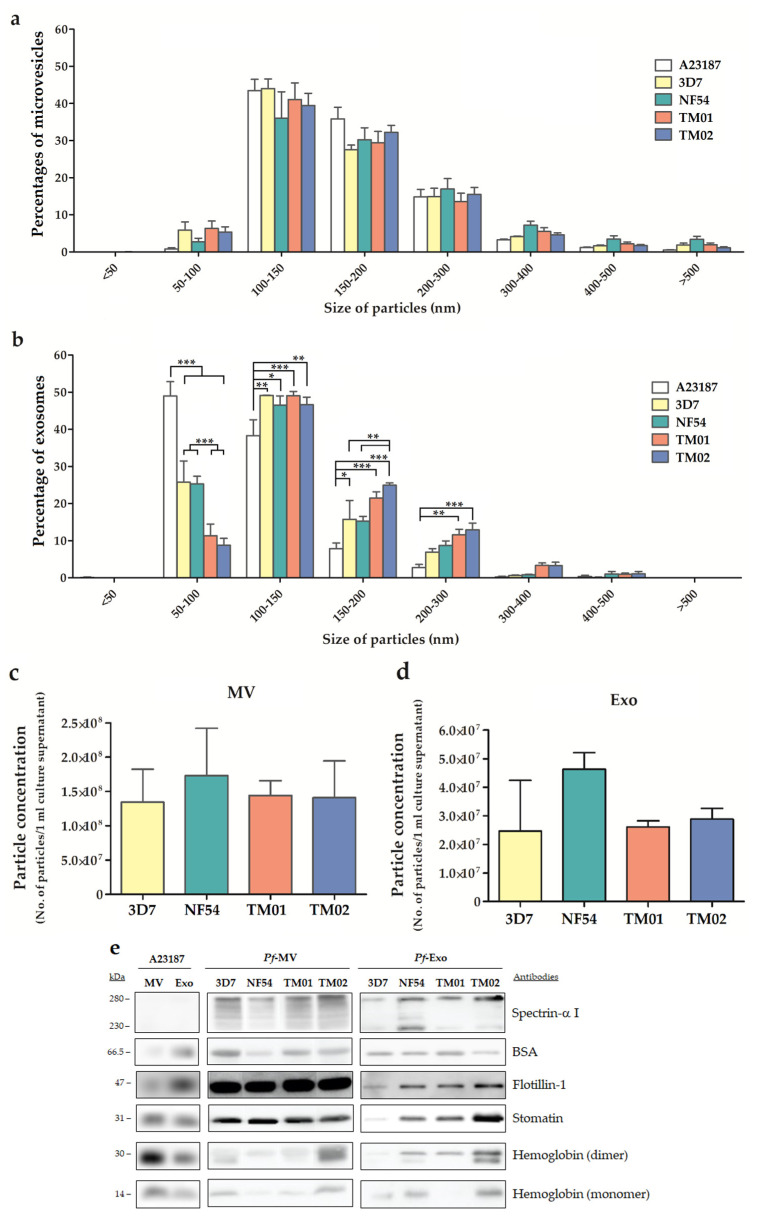
Characteristics of *Pf*-EVs derived from four *P. falciparum* strains (3D7, NF54, TM01, and TM02) and control EVs from A23187-activated RBCs. (**a**–**d**) NTA analysis. (**a**) Particle size distributions of control A23187-MV and *Pf*-MV. (**b**) Particle size distributions of control A23187-Exo and *Pf*-Exo. (**c**) Particle concentrations of *Pf*-MV. (**d**) Particle concentrations of *Pf*-Exo. (**e**) Representative Western blot analysis of A23187-EVs and *Pf*-EVs (MV and Exo). All samples were loaded at 10 µg total protein, except *Pf*-Exo were loaded at 5 µg total protein. Antibody markers and molecular weight as indicated. *n* = 2–5 for (**a**–**d**), *: *p* < 0.05, **: *p* < 0.01, and ***: *p* < 0.001.

The particle size distribution and numbers of particles per volume of culture supernatants of *Pf*-EVs, MV, and Exo from different *P. falciparum* strains were compared. The size of the majority population of control A23187-MV ranged from 100 to 300 nm, while the *Pf*-MV ranged from 50 to 400 nm populations (Figure 2a). The peaks of the *Pf*-MV sizes were 127.5 nm, 138.1 nm, 132.7 nm, and 135.8 nm for the 3D7, NF54, TM01, and TM02 strains, respectively. There were no statistical differences in the size distribution among these various MV populations. In parallel, the majority population of control A23187-Exo ranged from 50 to 150 nm in diameter, whereas the *Pf*-Exo were mainly within the 50–200 nm range (Figure 2b). The peaks of the *Pf*-Exo sizes were 110.3 nm, 102.1 nm, 114.9 nm, and 118.3 nm for the 3D7, NF54, TM01, and TM02 strains, respectively. These data showed that the size of *Pf*-Exo was larger than those of control A23187-Exo. The percentage within each size range of *Pf*-Exo and control A23187-Exo showed significant differences, especially in the 50–300 nm population. Interestingly, there were significant differences in the percentage of size distribution between *Pf*-Exo derived from 3D7 and NF54 compared to those from TM01 and TM02 in the 50–100 nm and 150–200 nm populations. These data suggested that a variation exists in the physical characteristics of *Pf*-Exo derived from different strains.

The concentrations of *Pf*-EVs from different *P. falciparum* strains were also measured by Nanoparticle Tracking Analysis (NTA) and compared. The number of EV particles/mL of the culture supernatant of *Pf*-MV was approximately five times higher than *Pf*-Exo in all *Pf*-EVs samples. The average number of *Pf*-MV and *Pf*-Exo isolated from one milliliter of malaria culture supernatant from the culture of 5% hematocrit and 5% malaria parasitemia ranged from 1.34 × 10^8^ to 1.73 × 10^8^ particles and 2.46 × 10^7^ to 4.63 × 10^7^ particles, respectively. However, there was no significant difference in the concentration of *Pf*-EV particles among the four *P. falciparum* strains (Figure 2c,d).

The Western blot analysis of the RBC proteins, commonly identified as RBC-EV markers, is shown in Figure 2e. The results showed that flotillin-1, cytosolic membrane-associated proteins, and stomatin, the integral membrane protein, were enriched in all EV samples. Moreover, hemoglobin, the RBC cytosolic protein, was enriched in all EVs, while spectrin, the RBC membrane skeleton marker, was found only in *Pf*-EVs. Although these *Pf*-EVs express all proteins. There was a variation in the levels of these proteins for each EV sample among the different *Pf* strains.

The results from the NTA and Western blot suggested that the differences in the physical characteristics of *Pf*-EVs from different *Pf* strains can be found in *Pf*-Exo but not *Pf*-MV.

### 2.2. Pf-EV Stimulation of Primary Monocytes

**Figure 3:**
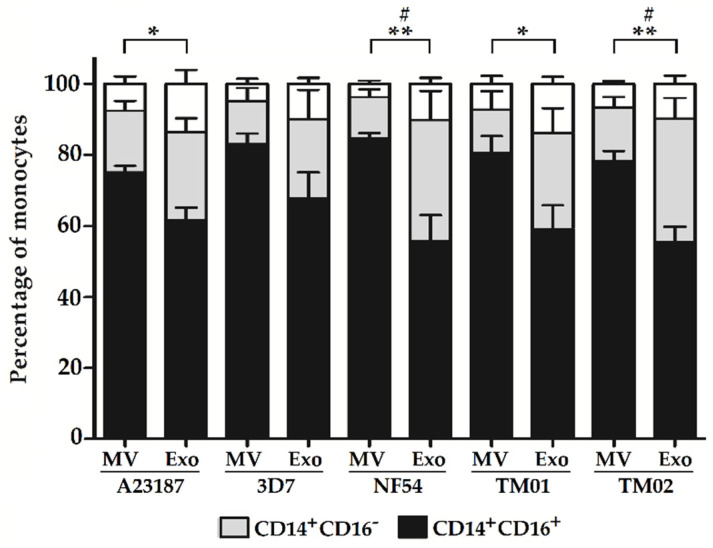
The percentage of monocyte subsets after EV stimulation: CD14^+^CD16^+^ monocyte (black bar), CD14^+^CD16^−^ monocyte (gray bar), CD14^dim^CD16+ monocyte, and CD14^−^ CD16^−^ cells (white bar). The data presented as mean ± SEM from 4 independent experiments. * *p* < 0.05 and ** *p* < 0.01 between *Pf*-MV and *Pf*-Exo stimulation in the CD14^+^CD16^+^ subset and # *p* < 0.05 between *Pf-*MV and *Pf*-Exo stimulation in the CD14^+^CD16^−^ subset.

Based on the immunophenotype, monocytes are classified into three subsets, which are CD14^+^CD16^−^ classical monocytes, CD14^+^CD16^+^ intermediate monocytes, and CD14^dim^ CD16^+^ nonclassical monocytes. Upon monocyte purification from freshly obtained PBMCs, the major cell populations from the isolated monocytes were classical monocytes. Of interest, the percentages of intermediate monocytes increased and became the major subpopulation after cell stimulation with all types of EVs. Although there were no differences in the frequency of all three cell subsets of *Pf*-MV-stimulated and *Pf*-Exo-stimulated cells among different *Pf* strains, the percentage of cell subsets stimulated with *Pf*-MV and *Pf*-Exo significantly differed. The frequencies of CD14^+^CD16^+^ intermediate monocytes stimulated with *Pf*-MV from the NF54, TM01, and TM02 strains were significantly higher than those from *Pf*-Exo stimulation (Figure 3, black bar). In parallel, the frequencies of CD14^+^CD16^−^ classical monocytes were also significantly lower in *Pf*-MV-stimulated cells compared to *Pf*-Exo-stimulated cells in the NF54 and TM02 strains (Figure 3, gray bar).

To explore the effect of *Pf*-EV stimulation on monocyte polarization, the cell surface expressions of CD80, CD86, CD163, and CD206 on the two major monocyte subpopulations, CD14^+^CD16^−^ classical monocytes and CD14^+^CD16^+^ intermediate monocytes, were further analyzed.

After stimulation with lipopolysaccharide (LPS)/ Interferon-γ (IFN-γ) and IL-4/IL-13 cytokines that are known to be the stimulators for M1-like and M2-like monocyte polarization, CD80 was expressed only on the LPS/IFN-γ-stimulated monocytes, whereas CD206 was upregulated on IL-4/IL-13-stimulated monocytes. Unexpectedly, the CD86 and CD163 expressions were detected in both unstimulated and stimulated cells. The representative flow cytometric analysis of cell surface marker expression is shown in Supplementary Figure S1. Therefore, based on the combinations of these surface receptor expressions, we classified the M1-like cells based on the expression of CD80 without CD206 (CD80^+^CD86^+^CD163^+^, CD80^+^CD86^+^, CD80^+^CD163^+^, and CD80^+^ cells), whereas the M2-like cells were the cells expressing CD206 without CD80 (CD86^+^CD163^+^CD206^+^, CD86^+^CD206^+^, CD163^+^CD206^+^, and CD206^+^ cells). In addition, the cells that expressed both CD80 and CD206 (CD80^+^CD86^+^CD163^+^CD206^+^, CD80^+^CD86^+^CD206^+^, CD80^+^CD163^+^CD206^+^, and CD80^+^CD206^+^ cells) were identified as mixed M1/M2-like cells and the cells without CD80 and CD206 expression (CD86^+^, CD86^+^CD163^+^, CD163^+^, and CD80^−^CD86^−^CD163^−^CD206^−^ cells) were classified as unpolarized cells.

**Figure 4:**
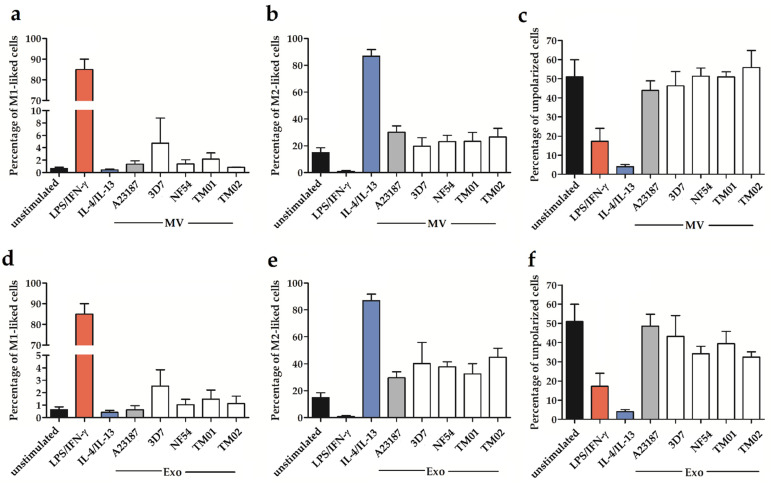
The monocyte polarization of the CD14^+^CD16^−^ subpopulation after EV stimulation: MV stimulation (upper panel) and Exo stimulation (lower panel). The percentage of M1-like cells (**a**,**d**), M2-like cells (**b**,**e**), and unpolarized cells (**c**,**f**) among the CD14^+^CD16^−^ subpopulation. LPS/IFN-γ and IL4/IL13 were stimulators for M1-like and M2-like cells. The data were shown as mean ± SEM from 4 independent experiments.

After cell stimulation with control A23187-EVs and *Pf*-EVs, the results of CD14^+^CD16^−^ monocytes showed that the baseline levels of M1-like cells in the unstimulated group were less than 1%, but the M2-like cells were as high as 20%. In addition, the stimulation with the control A23187-EVs and *Pf*-EVs, both MV and Exo, did not affect the levels of cell polarization, including M1-like cells (Figure 4a,d), M2-like cells (Figure 4b,e), and unpolarized cells (Figure 4c,f).

**Figure 5:**
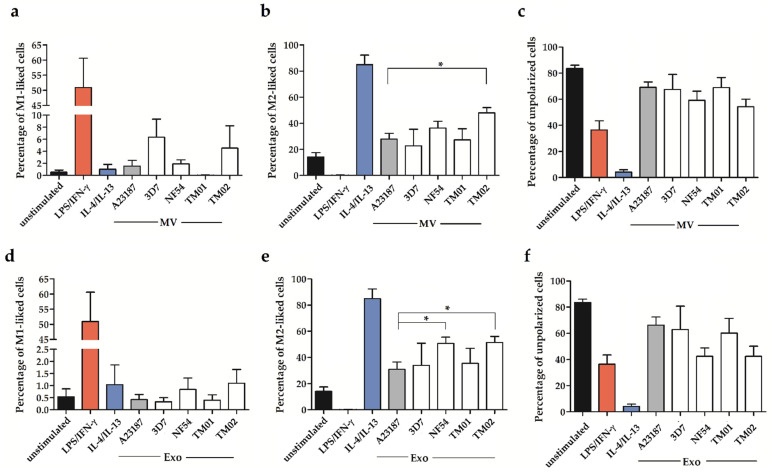
The monocyte polarization of the CD14^+^CD16^+^ subpopulation after EV stimulation: MV stimulation (upper panel) and Exo stimulation (lower panel). The percentage of M1-like cells (**a**,**d**), M2-like cells (**b**,**e**), and unpolarized cells (**c**,**f**) among the CD14^+^CD16^+^ subpopulation were shown. LPS/IFN-γ and IL4/IL13 were stimulators for M1-like and M2-like cells. The data showed as mean ± SEM from 4 independent experiments. * *p* < 0.05 between *Pf*-EV-stimulated cells and A23187-EV-stimulated cells.

To explore the CD14^+^CD16^+^ monocyte population, the baseline levels of M1-like and M2-like cells were similar to those in the CD14^+^CD16^−^ population. After EV stimulation, the percentages of M1-like, M2-like, and unpolarized cells of the control A23187-EV stimulation were comparable to those noted for unstimulated cells. Both *Pf*-MV and *Pf*-Exo did not affect the M1-like cell polarization (Figure 5a,d). However, the *Pf*-MV derived from *P. falciparum* strain TM02 significantly increased the percentage of M2-like cells (Figure 5b). Moreover, the *Pf*-Exo derived from *P. falciparum* strains NF54 and TM02 significantly increased M2-like cells compared to control A23187-Exo activation. The average percentages of M2-like cells were 50.72 ± 4.71, 51.28 ± 4.73, and 30.98 ± 5.67 in NF54-derived Exo-, TM02-derived Exo-, and control A23187 Exo-stimulated cells, respectively (Figure 5e). The mixed M1/M2 subpopulation was less than 3% in all activated groups. In parallel, the percentage of unpolarized cells seems to be decreased in the NF54-activated and TM02-activated cells (Figure 5f). These data indicate that *Pf*-EVs had a more pronounced effect on CD14^+^CD16^+^ than the CD14^+^CD16^−^ subpopulation, and the *Pf*-Exo derived from each *P. falciparum* strain might exhibit different monocyte polarization induction.

### 2.3. The miRNA Levels in Pf-EVs

Next, we further investigated the factor carried by *Pf*-EVs that might be involved in the M2-like polarization of *Pf*-EVs derived from the NF54 and TM02 strains. The miRNAs are one type of biomolecule carried by EVs, and several studies revealed that these miRNAs play an important role in intercellular communication and functional gene regulation in EV-targeted cells. Previous malaria-derived EVs studies showed that the *Plasmodium* spp. do not have their own miRNA machinery. Thus, the miRNAs found in *Pf*-EVs are only human miRNAs (hsa-miRNAs) [29]. According to the literature, the top miRNAs with the highest expression levels in erythrocytes include miR-451a, miR-144-3p, miR-16, miR-92a, let-7, and miR-486-5p [30]. The most abundant Argonaute 2-bound miRNAs in ex vivo stored RBCs are miR-16-5p, miR-451a-5p, miR-486-5p, and miR-92a-3p [31]. Two of these miRNAs, miR-486-5p and miR-451a, are also the most dominant miRNAs in peripheral blood [32]. In addition, the most abundant miRNAs in exosomes isolated from the supernatants of stored RBC units are miR-125b-5p, miR-4454, and miR-451a [33]. The miRNAs in RBC-EVs derived from the malaria culture are miR-451a, let-7b, miR-106b, miR-486, and miR-181a, and some of them traffic to and function in recipient cells [34,35]. Altogether, among these miRNAs, we further studied the validated levels of miR-451a, miR-486, miR-92a, and miR-16 in *Pf*-EVs, because they were highly expressed in erythrocytes and associated with increased hemolysis [36].

**Figure 6:**
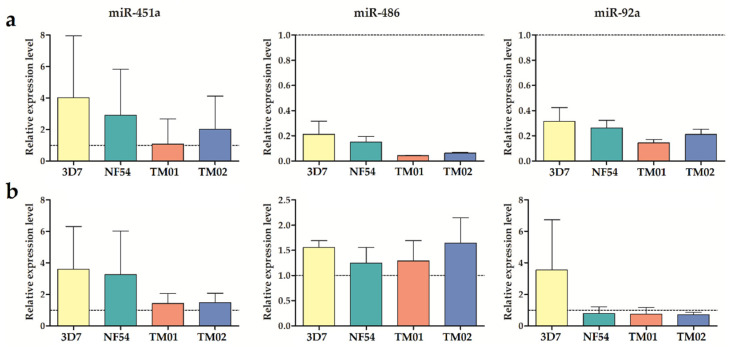
The relative miRNA expression levels of miR-451a, miR-486, and miR-92a in *Pf*-EVs. (**a**) *Pf*-MV and (**b**) *Pf*-Exo derived from 3D7, NF54, TM01, and TM02. The results were shown as mean ± SEM from three independent experiments. The lines indicate the levels of the control A23187-EVs.

Based on the normalization using the PCR-based Ct value of miR-16 that was used to internally normalize the level of red blood cell hemolysis for each *Pf*-EV sample, the results revealed that the miR-451a was carried in *Pf*-EVs at a high level compared to other miRNAs. Moreover, the levels of miR-451a within the *Pf*-EVs, both MV and Exo, seemed to be higher than in the control A23187-EVs. Interestingly, the levels of miR-486 were lower in *Pf*-MV when compared to the control A23187-MV, but their levels in *Pf*-Exo were higher than in the control A23187-Exo. In parallel, the miR-92a levels in *Pf*-MV also decreased compared to the control EVs, but their levels in *Pf*-Exo did not change. These data indicated that *P. falciparum* infection leads to the alteration of miRNAs in RBC EVs, and their miRNA levels were different in the *Pf*-EVs types. However, we did not find a significant difference in these three miRNA expressions among the *Pf*-EVs derived from *P. falciparum* strains (Figure 6). Thus, the miRNAs in *Pf*-EVs might not be a critical factor in driving M2-like monocyte polarization in *Pf*-EVs derived from TM02 strains. Further investigations are therefore warranted to address this issue.

## 3. Discussion

The influence of intraspecies variation, which includes the genomic and phenotypic diversity, among pathogenic strains might impact the growth development and virulence of the pathogen, the host immune response during infection, and the clinical output of the disease. For more than two decades, parasite-derived EVs have been studied for their role in virulence, infectivity, and modulation of the immune response during infection. The present study investigated the intraspecies variation of *Pf*-EV characteristics among the *Pf* strains. Firstly, the *Pf*-EVs were isolated from the *Pf* culture supernatant of various strains, and the physical characteristics of these *Pf*-EVs were compared. The previous study by Vimonpatranon S et al. [37] documented the efficient EV isolation protocol from *Pf* culture media, and the morphology and the average size of *Pf*-MV and *Pf*-Exo were reported. This study also adds more information on the particle size distribution of *Pf*-EVs derived from those *Pf* strains based on the same protocols for malarial cultures and EV isolation. We found that the majority population of *Pf*-MV and *Pf*-Exo ranged from 50 to 400 nm and 50 to 200 nm, respectively. Our data also paralleled the previous study by Opadokun T et al. [38], who reported that the majority of *Pf*-EVs were in the 100–300 nm range. There were slight differences that might be due to the difference in the protocols of parasite cultures and *Pf*-EV isolation. We also found the variations in the *Pf*-Exo size distribution among different *Pf* strains. This particle size variation might affect the uptake rate by target cells and their effectiveness in cellular response induction on target cells.

As monocytes/macrophages play a role in eliminating *P. falciparum*-iRBCs, monocyte activation by parasite products during malaria infection has been reported. For example, the hemozoin malarial pigment was shown to trigger the overproduction of TNF-α, IL-1β, and MIP-1α from human monocytes by activating the p38 MAPK and NF-κB pathways [39]. The immune complexes of parasite DNA and anti-DNA antibodies induce the assembly of inflammasome, caspase-1 activation, and cytokine production by the CD14^+^CD16^+^CD64^hi^CD32^low^ monocyte of patients with either *P. falciparum* or *P. vivax* malaria [40]. The iRBC-derived MV, which is highly released at a late stage during erythrocytic schizogony, activated proinflammatory and anti-inflammatory cytokine production by human macrophages and induced neutrophil migration [26]. The plasmodial DNA delivery by *Pf*-EVs also induces monocyte activation and type 1 IFN production through a STING-dependent pathway [27]. In this study, we also found that *Pf*-EVs could activate the monocyte to change the subset from the classical monocyte to the intermediate monocyte subset. This effect was found in both control A23187-EVs and *Pf*-EVs stimulation and was more pronounced in MV stimulation than in Exo stimulation. A previous study showed that smaller exosomes were taken up faster in glioblastoma cells [41]. In addition, the sialylated N-glycans carried by EVs are essential for EV uptake by human monocytes. The *Pf*-EVs contained significantly higher sialylated complex N-glycans than those on uninfected RBC-derived EVs [28]. Thus, the effective uptake and cellular response to EVs might be varied depending on the characteristics of the EVs and the target cell types. However, this study did not evaluate the variations in EV uptake between monocyte subsets and EV types. Nevertheless, this stimulated effect does not seem to affect the monocyte polarization, as we cannot detect the alteration of the M1/M2 monocyte subset in control A23187 EV-stimulated groups.

Monocyte polarization is a complex process regulated by various activating molecules, intracellular transcription factors, and downstream signaling pathways. EV-induced macrophage polarization has been described in many diseases [42]. *P. falciparum-*infected red blood cell lysate induces the epigenetic reprogramming of monocytes/macrophages toward a regulatory phenotype that attenuates inflammatory responses during the subsequent *P. falciparum* exposure [43]. Our results also showed that the *Pf*-EVs derived from the *Pf* TM02 strain induced a higher percentage of M2-like intermediate monocytes when compared to other strains. This potency might be another virulent factor for this *Pf* strain.

The EV contents, for example, miRNAs [44,45,46], cytokines such as monocyte chemoattractant protein-1 (MCP-1), and IL-10 [47,48], and proteins such as tumor necrosis factor-α-stimulated gene/protein-6 (TSG-6), tumor necrosis factor-related apoptosis-inducing ligand (TRAIL), and STAT3 [49,50,51] in cell-derived EVs have been shown to be active stimulators mediating in monocyte polarization in EV-recipient cells.

As miRNAs are one of the most widely studied biomolecules carried by EVs, several studies have revealed their roles in monocyte/macrophage differentiation. For example, the exosomes derived from mesenchymal stem cells (MSCs) induce macrophage polarization toward the macrophage M2 phenotype by regulating via exosome-derived miR-223 targeting Pknox1 [52]. In addition, MSC-derived EV-miR-182 and LPS-preconditioned MSC-derived EV-let-7b regulate macrophage M2-like polarization by mediating via the TLR4/NF-κB/STAT3/AKT signaling pathway, which leads to reduced inflammation [53,54].

Although the miRNAs contained in RBCs and RBC-EVs are not the primary miRNAs involved in monocyte activation and polarization, some of them can target specific genes related to the responsiveness of monocytes. We found that *Pf*-MV and *Pf*-Exo carried different levels of miRNAs in RBC-EVs. As expected, miR-451a was highly expressed in *Pf*-EVs compared to the other miRNAs. miR-451a directly targets and regulates many proteins, such as the YWHAZ/14-3-3ζ protein, which is associated with the suppression of type I IFN, IL-6, TNF-α, and RANTES expression, and CXCL16, which is the chemokine responsible for the recruitment of monocytes and tumor-associated macrophage differentiation [55,56,57]. miR-486-5p directly targets HAT1 and is significantly correlated with the expression of IL-6, IL-8, TNF-α, and IFN-γ [58]. Overexpression of miR-486 in myeloid cells promoted the proliferation and suppressed apoptosis and differentiation of the cells [59]. The increasing level of miR-92a decreased the activation of the JNK/c-Jun pathway and the production of inflammatory cytokines in the macrophages with TLR4 stimulation [60]. In addition, PI3K/Akt signaling mediating via mTORC1 regulates the effector responses of macrophages and has a direct effect on promoting M2 macrophage polarization [61,62,63]. The signaling genes in this pathway were also the targets of miR-16-5p, miR-92a-3p, miR-451a, and miR-486-5p [64,65]. Thus, we speculated that these high potential miRNAs in *Pf*-EVs might contribute to M2 polarization in highly virulent *P. falciparum* strains. However, our data did not show any difference in the levels of these miRNAs among *Pf*-EVs derived from different strains. Therefore, these miRNAs might not be a significant factor as previously thought in driving the M2-like polarization of TM02-derived *Pf*-EVs.

Almost all miRNAs in mature RBCs are active, which present as a miRNA-RISC complex. *Plasmodium* spp. infection might influence the binding of host miRNAs on parasite target genes and the sorting of selected miRNAs and loading them into EVs. Therefore, the level of miRNAs in *Pf-*EVs might directly reflect the miRNA content in iRBCs. Although the mechanisms of miRNA loading in RBC-EV have not been extensively studied, our results suggest that there are differences in the miRNA loading capacity between EV types.

Our previous study explored the protein contents in *Pf*-EVs using a proteomic approach. We found that *Pf*-EVs contain both human and *Plasmodium* spp. proteins. These parasite proteins might be a factor that can induce monocyte activation. Furthermore, many human proteins carried by *Pf*-Exo are also functionally enriched in innate immune response pathways, such as Interleukin-1 receptor-associated kinase 4 (IRAK-4), Nuclear factor NF-kappa-B p105 subunit (NFKB1), and DNA-dependent protein kinase catalytic subunit (PRKDC) [37]. These proteins in EVs might be another factor driving monocyte polarization. Thus, the monocyte polarization of the virulent *Pf* strain might be the overall net outcome of the activation effects of biomolecules in *Pf*-EVs, including miRNAs; proteins; and other critical factors such as parasite DNA, RNA, and metabolites.

Future studies should explore in depth the target genes, proteins, and signaling pathways of *Pf*-EVs derived from the *P. falciparum* strain with high virulence or severe clinical complications that induce M2-like polarization. The results might add to the knowledge that EVs modulate the host immune response, leading to increased pathogenesis.

## 4. Materials and Methods

### 4.1. Ethical Approved

The study was conducted following the Declaration of Helsinki, and the Siriraj Institutional Review Board of the Faculty of Medicine Siriraj Hospital, Mahidol University, Bangkok, Thailand, approved the protocols (COA No. Si632/2017).

### 4.2. Plasmodium falciparum Culture

The whole blood was collected from healthy donors in acid-citrate-dextrose (ACD) anticoagulant (Gibco, Grand Island, NY, USA). The plasma was isolated from the whole blood, and the packed red cells were washed and resuspended in complete RPMI media [(RPMI 1640 (Gibco) supplemented with 25 mM HEPES (Sigma-Aldrich, St. Louis, MO, USA), 0.225% NaHCO_3_ (Sigma-Aldrich), 0.1 mM hypoxanthine (Sigma-Aldrich), 25 g/mL gentamicin (Sigma-Aldrich), and 5% AlbuMAX™ II (Gibco; Auckland, New Zealand)]. The fresh red blood cells were kept at 4 °C and used for malaria culture within a week.

Four *P. falciparum* strains (3D7, NF54, TM01, and TM02) were used in the study. The *P. falciparum* NF54 is a strain isolated from a patient with uncomplicated malaria, and *P. falciparum* 3D7 is a clone of the *P. falciparum* strain NF54 [66]. The 3D7 and NF54 are pan-antimalarial drug-sensitive strains. They are susceptible to chloroquine, mefloquine, piperaquine, dihydroartemisinin, cycloguanil, and pyrimethamine but resistant to sulfadoxine [67,68,69]. The *P. falciparum* strains TM01 and TM02 were culture-adapted isolates collected from uncomplicated and severe *P. falciparum* malaria patients in Thailand, respectively. The TM01 and TM02 are chloroquine resistant (IC_50_ 100−200 ng/mL) and artemisinin-sensitive parasites (IC_50_ 1−2 ng/mL).

The *P. falciparum* strains were continuously cultured in 5% hematocrit of human red blood cells in complete RPMI media in a 5% CO_2_ plus 5% O_2_. The parasite culture pellet was resuspended in 5% D-sorbitol solution (Sigma-Aldrich) for 10 min at 37 °C to prepare the ring-staged parasites. Next, the synchronized ring-stage parasites were cultured at 5% parasitemia in 5% hematocrit for 24 h. Then, the culture media was removed, and the new culture media was added to the immature-trophozoite parasite culture and cultured for 24 h. The culture media during the growth development from immature-trophozoite parasites to ring-staged parasites was collected and centrifuged at 1500× *g* for 15 min to remove the red blood cells and cell debris and kept at −20 °C until used for EVs isolation.

### 4.3. EVs Isolation and Characterization

As previously described, the *Pf*-EVs were isolated from malarial culture media by multi-step centrifugation [37]. First, the 400 mL malarial culture media was centrifuged at 13,000× *g* for 2 min and subsequently filtered by passing through a 1.2 µm filter. Next, the filtered culture media was centrifuged at 21,000× *g* (Thermo Scientific™ Sorvall RC 6 Plus superspeed centrifuge; Waltham, MA, USA) for 70 min at 4 °C to collect the MV pellet. Then, the 90% upper part of the supernatant was further filtered through a 0.2 µm membrane filter and centrifuged at 110,000× *g* (Thermo Scientific™ Sorvall WX80 ultracentrifuge; Waltham, MA, USA) for 90 min at 4 °C to collect the Exo pellet. Finally, the EV pellets were washed with 0.2 µm-filtrated phosphate buffer saline (PBS) and stored at –80 °C until use. The control EVs were separated from the culture supernatant of 10 µM A23187-activated uninfected RBCs at 20% hematocrit for 24 h, and the culture supernatant was collected and used to isolate A23187-MV and A23187-Exo as same as *Pf*-EVs.

### 4.4. Assessment of EVs Morphology by Electron Microscopy

The morphology of *Pf*-MV and *Pf*-Exo was visualized by FE-SEM and TEM, respectively. For FE-SEM, the specimen stub was sputter-coated with a thin layer of gold using a sputter coater [70]. Then, *Pf*-MV morphology was observed by Nova NanoSEM™450 (Thermo Fisher, Waltham, MA, USA) at an accelerating voltage of 10 kV. For TEM, the *Pf*-Exo was fixed with paraformaldehyde solution, stained with uranyl acetate, and the sample was imaged using an FEI Tecnai T12 Transmission Electron Microscope (Field Electron and Ion Co., Hillsboro, OR, USA) operated at 100 kV following the protocol as previously described [37].

### 4.5. Nanoparticle Tracking Analysis (NTA)

To characterize the size distribution and concentration of EV particles, the isolated EVs were diluted with 0.2 µm-filtered PBS at the appropriate dilution, making it approximately 20−100 particles per frame, according to the instrument’s recommendation. Then, the samples were injected into the NanoSight NS300 Instrument (Malvern Panalytical Ltd., Worcester, UK) using a Nanosight syringe pump. The standard operation procedure (SOP) used for data acquisition is as follows: the camera level at 13, particles per frame at 20−100, the capture of five sequential 1 min videos, and the detector threshold at 5. Data analysis was performed using NTA 3.4 software (Malvern Panalytical Ltd., Worcester, UK).

### 4.6. Western Blotting Analysis

The protein concentrations of control A23187-EVs and *Pf*-EVs were determined by the Bradford protein assay (Bio-Rad Laboratories Ltd.; Hercules, CA, USA) according to the manufacturer’s protocols. The 5–10 µg of isolated *Pf*-EVs were run on 12% SDS-polyacrylamide gel electrophoresis, and the proteins were transferred to polyvinylidene difluoride membranes. The membranes were blocked with 5% nonfat milk in Tris-buffered saline containing 0.1% Tween20 (TBST), and the blots were probed with primary antibodies, including flotillin-1 (clone C-2, Santa Cruz Biotechnology, Inc., Dallas, TX, USA), stomatin (clone E-5, Santa Cruz Biotechnology, Inc.), spectrin ⍺ I (clone B-12, Santa Cruz Biotechnology, Inc.), hemoglobin β/γ/δ/ε (clone A-8, Santa Cruz Biotechnology, Inc.), and BSA (clone 25G7, Thermo Fisher Scientific, Waltham, MA USA) in blocking buffer in TBST for 1 h at room temperature. Then the blots were washed with TBST and incubated with either goat anti-mouse PER-conjugated horseradish peroxidase (ImmunoTools GmbH; Friesoythe, Germany) or mouse IgG Fc binding protein conjugated to horseradish peroxidase (m-IgG Fc BP-HRP, Santa Cruz Biotechnology, Inc.) for 1 h at room temperature. After washing, the Clarity™ Western ECL substrate was added (Bio-Rad Laboratories Ltd.), and the signal was detected by ImageQuant™ LAS 4010 (GE Healthcare; Boston, CA, USA). For antibody re-probing, the membrane was detected with anti-flotillin-1, anti-stomatin, anti-spectrin ⍺ I, and anti-hemoglobin β/g/d/e, respectively.

### 4.7. miRNA Determination

According to the manufacturer’s instructions, total miRNAs were extracted from *Pf*-EVs by the miRNeasy Mini Kit (Qiagen, Hilden, Germany). The RNA’s quality and quantity were examined by a Nanodrop-8000 Spectrophotometer (Thermo Scientific, Wilmington, DE, USA). The cDNA was synthesized using the miScript II RT Kit (Qiagen) and miScript HiSpec Buffer. Then, the levels of the miRNA expressions were quantitatively analyzed by real-time PCR using miScript primer assays (Qiagen) and miScript SYBR Green PCR kits (Qiagen) on the LightCycler^®^ 480 System (Roche Diagnostics GmbH, Mannheim, Germany). Finally, the levels of the miRNA expressions were analyzed using the ^ΔΔCT^ method normalized to the expression of hsa-miR-16 [71,72,73]. The 2^−ΔΔCT^-method was used to calculate the fold change relative to the corresponding expression level of each miRNA in the control A23187-EVs. The assay details were shown in Table 1.

### 4.8. Primary Monocyte Isolation

The heparinized venous whole blood was collected from healthy donors who had no previous history of malaria infection. The peripheral mononuclear cells (PBMCs) were isolated by the Ficoll–Hypaque density gradient centrifugation using Histopaque^®^-1077 (Sigma-Aldrich). The primary monocytes were purified from PBMCs by negative selection magnetic cell separation using the MojoSort™ Human Pan Monocyte Isolation Kit (Biolegend, San Diego, CA, USA). The viability of isolated monocytes was measured by Trypan blue exclusion with a TC10™ automated cell counter (Bio-Rad Laboratories, Inc.). To determine the purity of the isolated monocytes, 1 × 10^5^-purified monocytes were incubated with Human Trustain FcX™ (Biolegend) for 10 min, followed by staining with the combination of fluorochrome-labeled specific antibodies, including allophycocyanin (APC)-conjugated-CD14 (clone HCD14; Biolegend) and fluorescein isothiocyanate (FITC)-conjugated-CD16 (clone 3G8; BD Pharmingen™, San Diego, CA, USA) at 4 °C for 30 min. The stained cells were analyzed by a BD^®^LSRII flow cytometer (BD Bioscience; San Diego, CA, USA) using FACSDiva^TM^ software Version 7 (BD Biosciences). The purity and percentage of the monocyte subsets were analyzed by FlowJo™ software Version 10 (Becton, Dickinson and Company, Ashland, OR, USA). More than 85% purity was used in the experiment.

### 4.9. Coculture of Pf-EVs with Primary Monocytes

The 3 × 10^5^ purified monocytes were cultured with either 3 × 10^9^ particles of *Pf*-EVs or control A23187-EVs in a 24-well plate for 60 h in 5% CO_2_ plus 5% O_2_. The combination of either 50 ng/mL LPS from *Escherichia coli* (Sigma-Aldrich) with 10 ng/mL recombinant human IFN-γ (Sigma-Aldrich) or 100 ng/mL recombinant human IL-4 (ImmunoTools GmbH) with 100 ng/mL recombinant human IL-13 (ImmunoTools GmbH) were used as control activators for M1-like and M2-like monocyte polarization, respectively.

After stimulation, the monocytes were stained with Zombie Aqua™ Fixable Viability dye (Biolegend), followed by incubation with Human Trustain FcX™ (Biolegend) for 10 min. Then, the monocytes were stained with a combination of fluorescent-labeled antibodies, including APC-conjugated-CD14 (clone HCD14; Biolegend), FITC-conjugated-CD16 (clone 3G8; BD Pharmingen™), Phycoerythrin (PE)-conjugated-CD80 (clone 2D10; Biolegend), PE/Dazzle-conjugated-CD86 (clone 2331; Biolegend), Brilliant Violet™421 (BV421)-conjugated-CD163 (clone GHI/63; Biolegend), and Alexa Fluor^®^700-conjugated-CD206 (clone 15-2; Biolegend) at 4 °C for 30 min. The fluorescent-labeled isotype-matched antibodies (clone MOPC-21; Biolegend) were used as staining controls. The stained cells were analyzed by the BD LSRFortessa™ Cell Analyzer (BD Bioscience) using FACSDiva™ software Version 7 (BD Bioscience). A post-acquisition analysis was performed using FlowJo™ software version 10 (Becton, Dickinson and Company).

### 4.10. Statistical Analysis

Data were analyzed using GraphPad Prism software (GraphPad Software Inc., San Jose, CA, USA). The comparisons between the activated and control groups were performed using unpaired *t*-tests. Group differences with *p*-values less than 0.05 were considered statistically significant.

## Figures and Tables

**Table 1:** Primers used for quantitative PCR.

miRBase ID	miRBase Accession	Mature miRNA Sequence
hsa-miR-451a	MIMAT0001631	5’AAACCGUUACCAUUACUGAGUU
hsa-miR-486-5p	MIMAT0002177	5’UCCUGUACUGAGCUGCCCCGAG
hsa-miR-92a-3p	MIMAT0000092	5’UAUUGCACUUGUCCCGGCCUGU
hsa-miR-16-5p	MIMAT0000069	5’UAGCAGCACGUAAAUAUUGGCG

## Data Availability

Not applicable.

## Supplementary Materials

The following supporting information can be downloaded at: https://www.mdpi.com/article/10.3390/ijms24032631/s1:

## References

[1] WHO (2022). World Malaria Report 2022.

[2] Overstreet M.G., Cockburn I.A., Chen Y.C., Zavala F. (2008). Protective CD8 T cells against Plasmodium liver stages: Immunobiology of an ‘unnatural’ immune response. Immunol. Rev..

[3] Riley E.M., Stewart V.A. (2013). Immune mechanisms in malaria: New insights in vaccine development. Nat. Med..

[4] Ortega-Pajares A., Rogerson S.J. (2018). The Rough Guide to Monocytes in Malaria Infection. Front. Immunol..

[5] Chua C.L., Brown G., Hamilton J.A., Rogerson S., Boeuf P. (2013). Monocytes and macrophages in malaria: Protection or pathology?. Trends Parasitol..

[6] Wong K.L., Yeap W.H., Tai J.J., Ong S.M., Dang T.M., Wong S.C. (2012). The three human monocyte subsets: Implications for health and disease. Immunol. Res..

[7] Dobbs K.R., Embury P., Vulule J., Odada P.S., Rosa B.A., Mitreva M., Kazura J.W., Dent A.E. (2017). Monocyte dysregulation and systemic inflammation during pediatric falciparum malaria. JCI Insight.

[8] Loughland J.R., Woodberry T., Field M., Andrew D.W., SheelaNair A., Dooley N.L., Piera K.A., Amante F.H., Kenangalem E., Price R.N. (2020). Transcriptional profiling and immunophenotyping show sustained activation of blood monocytes in subpatent Plasmodium falciparum infection. Clin. Transl. Immunol..

[9] Chimma P., Roussilhon C., Sratongno P., Ruangveerayuth R., Pattanapanyasat K., Perignon J.L., Roberts D.J., Druilhe P. (2009). A distinct peripheral blood monocyte phenotype is associated with parasite inhibitory activity in acute uncomplicated Plasmodium falciparum malaria. PLoS Pathog..

[10] Zhou J., Feng G., Beeson J., Hogarth P.M., Rogerson S.J., Yan Y., Jaworowski A. (2015). CD14(hi)CD16+ monocytes phagocytose antibody-opsonised Plasmodium falciparum infected erythrocytes more efficiently than other monocyte subsets, and require CD16 and complement to do so. BMC Med..

[11] Royo J., Rahabi M., Kamaliddin C., Ezinmegnon S., Olagnier D., Authier H., Massougbodji A., Alao J., Ladipo Y., Deloron P. (2019). Changes in monocyte subsets are associated with clinical outcomes in severe malarial anaemia and cerebral malaria. Sci. Rep..

[12] Wang N., Liang H., Zen K. (2014). Molecular mechanisms that influence the macrophage m1-m2 polarization balance. Front. Immunol..

[13] Murray P.J. (2017). Macrophage Polarization. Annu. Rev. Physiol..

[14] Orecchioni M., Ghosheh Y., Pramod A.B., Ley K. (2019). Macrophage Polarization: Different Gene Signatures in M1(LPS+) vs. Classically and M2(LPS-) vs. Alternatively Activated Macrophages. Front. Immunol..

[15] Wei Y., Schober A. (2016). MicroRNA regulation of macrophages in human pathologies. Cell. Mol. Life Sci..

[16] Essandoh K., Li Y., Huo J., Fan G.C. (2016). MiRNA-Mediated Macrophage Polarization and its Potential Role in the Regulation of Inflammatory Response. Shock.

[17] Weinberg J.B., Volkheimer A.D., Rubach M.P., Florence S.M., Mukemba J.P., Kalingonji A.R., Langelier C., Chen Y., Bush M., Yeo T.W. (2016). Monocyte polarization in children with falciparum malaria: Relationship to nitric oxide insufficiency and disease severity. Sci. Rep..

[18] Mandala W.L., Msefula C.L., Gondwe E.N., Drayson M.T., Molyneux M.E., MacLennan C.A. (2016). Monocyte activation and cytokine production in Malawian children presenting with *P. falciparum* malaria. Parasite Immunol..

[19] Parroche P., Lauw F.N., Goutagny N., Latz E., Monks B.G., Visintin A., Halmen K.A., Lamphier M., Olivier M., Bartholomeu D.C. (2007). Malaria hemozoin is immunologically inert but radically enhances innate responses by presenting malaria DNA to Toll-like receptor 9. Proc. Natl. Acad.Sci. USA.

[20] Krishnegowda G., Hajjar A.M., Zhu J., Douglass E.J., Uematsu S., Akira S., Woods A.S., Gowda D.C. (2005). Induction of proinflammatory responses in macrophages by the glycosylphosphatidylinositols of Plasmodium falciparum: Cell signaling receptors, glycosylphosphatidylinositol (GPI) structural requirement, and regulation of GPI activity. J. Biol. Chem..

[21] Couper K.N., Barnes T., Hafalla J.C., Combes V., Ryffel B., Secher T., Grau G.E., Riley E.M., de Souza J.B. (2010). Parasite-derived plasma microparticles contribute significantly to malaria infection-induced inflammation through potent macrophage stimulation. PLoS Pathog..

[22] Sampaio N.G., Eriksson E.M., Schofield L. (2018). Plasmodium falciparum PfEMP1 Modulates Monocyte/Macrophage Transcription Factor Activation and Cytokine and Chemokine Responses. Infect. Immun..

[23] Campos F.M., Franklin B.S., Teixeira-Carvalho A., Filho A.L., de Paula S.C., Fontes C.J., Brito C.F., Carvalho L.H. (2010). Augmented plasma microparticles during acute Plasmodium vivax infection. Malar. J..

[24] Pankoui Mfonkeu J.B., Gouado I., Fotso Kuate H., Zambou O., Amvam Zollo P.H., Grau G.E., Combes V. (2010). Elevated cell-specific microparticles are a biological marker for cerebral dysfunctions in human severe malaria. PLoS ONE.

[25] Yanez-Mo M., Siljander P.R., Andreu Z., Zavec A.B., Borras F.E., Buzas E.I., Buzas K., Casal E., Cappello F., Carvalho J. (2015). Biological properties of extracellular vesicles and their physiological functions. J. Extracell. Vesicles.

[26] Mantel P.Y., Hoang A.N., Goldowitz I., Potashnikova D., Hamza B., Vorobjev I., Ghiran I., Toner M., Irimia D., Ivanov A.R. (2013). Malaria-infected erythrocyte-derived microvesicles mediate cellular communication within the parasite population and with the host immune system. Cell Host Microbe.

[27] Sisquella X., Ofir-Birin Y., Pimentel M.A., Cheng L., Abou Karam P., Sampaio N.G., Penington J.S., Connolly D., Giladi T., Scicluna B.J. (2017). Malaria parasite DNA-harbouring vesicles activate cytosolic immune sensors. Nat. Commun..

[28] Hila Ben Ami Pilo S.K.K., Lühle J., Biskup K., Gal B.L., Goldian I.R., Alfandari D., Revach O.-Y., Kiper E., Rotkopf R., Porat Z. (2022). Sialylated N-glycans mediate monocyte uptake of extracellular vesicles secreted from Plasmodium falciparum-infected red blood cells. J. Extracell. Biol..

[29] Xue X., Zhang Q., Huang Y., Feng L., Pan W. (2008). No miRNA were found in Plasmodium and the ones identified in erythrocytes could not be correlated with infection. Malar. J..

[30] Doss J.F., Corcoran D.L., Jima D.D., Telen M.J., Dave S.S., Chi J.T. (2015). A comprehensive joint analysis of the long and short RNA transcriptomes of human erythrocytes. BMC Genom..

[31] Vu L., Ragupathy V., Kulkarni S., Atreya C. (2017). Analysis of Argonaute 2-microRNA complexes in ex vivo stored red blood cells. Transfusion.

[32] Jee D., Yang J.S., Park S.M., Farmer D.T., Wen J., Chou T., Chow A., McManus M.T., Kharas M.G., Lai E.C. (2018). Dual Strategies for Argonaute2-Mediated Biogenesis of Erythroid miRNAs Underlie Conserved Requirements for Slicing in Mammals. Mol. Cell.

[33] Huang H., Zhu J., Fan L., Lin Q., Fu D., Wei B., Wei S. (2019). MicroRNA Profiling of Exosomes Derived from Red Blood Cell Units: Implications in Transfusion-Related Immunomodulation. Biomed. Res. Int..

[34] Mantel P.Y., Hjelmqvist D., Walch M., Kharoubi-Hess S., Nilsson S., Ravel D., Ribeiro M., Gruring C., Ma S., Padmanabhan P. (2016). Infected erythrocyte-derived extracellular vesicles alter vascular function via regulatory Ago2-miRNA complexes in malaria. Nat. Commun..

[35] Wang Z., Xi J., Hao X., Deng W., Liu J., Wei C., Gao Y., Zhang L., Wang H. (2017). Red blood cells release microparticles containing human argonaute 2 and miRNAs to target genes of Plasmodium falciparum. Emerg. Microbes Infect..

[36] Pritchard C.C., Kroh E., Wood B., Arroyo J.D., Dougherty K.J., Miyaji M.M., Tait J.F., Tewari M. (2012). Blood cell origin of circulating microRNAs: A cautionary note for cancer biomarker studies. Cancer Prev. Res..

[37] Vimonpatranon S., Roytrakul S., Phaonakrop N., Lekmanee K., Atipimonpat A., Srimark N., Sukapirom K., Chotivanich K., Khowawisetsut L., Pattanapanyasat K. (2022). Extracellular Vesicles Derived from Early and Late Stage Plasmodium falciparum-Infected Red Blood Cells Contain Invasion-Associated Proteins. J. Clin. Med..

[38] Opadokun T., Agyapong J., Rohrbach P. (2022). Protein Profiling of Malaria-Derived Extracellular Vesicles Reveals Distinct Subtypes. Membranes.

[39] Polimeni M., Valente E., Aldieri E., Khadjavi A., Giribaldi G., Prato M. (2012). Haemozoin induces early cytokine-mediated lysozyme release from human monocytes through p38 MAPK- and NF-kappaB-dependent mechanisms. PLoS ONE.

[40] Hirako I.C., Gallego-Marin C., Ataide M.A., Andrade W.A., Gravina H., Rocha B.C., de Oliveira R.B., Pereira D.B., Vinetz J., Diamond B. (2015). DNA-Containing Immunocomplexes Promote Inflammasome Assembly and Release of Pyrogenic Cytokines by CD14+ CD16+ CD64high CD32low Inflammatory Monocytes from Malaria Patients. mBio.

[41] Caponnetto F., Manini I., Skrap M., Palmai-Pallag T., Di Loreto C., Beltrami A.P., Cesselli D., Ferrari E. (2017). Size-dependent cellular uptake of exosomes. Nanomedicine.

[42] Tang D., Cao F., Yan C., Fang K., Ma J., Gao L., Sun B., Wang G. (2022). Extracellular Vesicle/Macrophage Axis: Potential Targets for Inflammatory Disease Intervention. Front. Immunol..

[43] Guha R., Mathioudaki A., Doumbo S., Doumtabe D., Skinner J., Arora G., Siddiqui S., Li S., Kayentao K., Ongoiba A. (2021). Plasmodium falciparum malaria drives epigenetic reprogramming of human monocytes toward a regulatory phenotype. PLoS Pathog..

[44] Shen D., He Z. (2021). Mesenchymal stem cell-derived exosomes regulate the polarization and inflammatory response of macrophages via miR-21-5p to promote repair after myocardial reperfusion injury. Ann. Transl. Med..

[45] He S., Wu C., Xiao J., Li D., Sun Z., Li M. (2018). Endothelial extracellular vesicles modulate the macrophage phenotype: Potential implications in atherosclerosis. Scand J. Immunol..

[46] Li J., Xue H., Li T., Chu X., Xin D., Xiong Y., Qiu W., Gao X., Qian M., Xu J. (2019). Exosomes derived from mesenchymal stem cells attenuate the progression of atherosclerosis in ApoE(-/-) mice via miR-let7 mediated infiltration and polarization of M2 macrophage. Biochem. Biophys. Res. Commun..

[47] Lv L.L., Feng Y., Wen Y., Wu W.J., Ni H.F., Li Z.L., Zhou L.T., Wang B., Zhang J.D., Crowley S.D. (2018). Exosomal CCL2 from Tubular Epithelial Cells Is Critical for Albumin-Induced Tubulointerstitial Inflammation. J. Am. Soc. Nephrol..

[48] Tang T.T., Wang B., Wu M., Li Z.L., Feng Y., Cao J.Y., Yin D., Liu H., Tang R.N., Crowley S.D. (2020). Extracellular vesicle-encapsulated IL-10 as novel nanotherapeutics against ischemic AKI. Sci. Adv..

[49] An J.H., Li Q., Ryu M.O., Nam A.R., Bhang D.H., Jung Y.C., Song W.J., Youn H.Y. (2020). TSG-6 in extracellular vesicles from canine mesenchymal stem/stromal is a major factor in relieving DSS-induced colitis. PLoS ONE.

[50] Hirsova P., Ibrahim S.H., Krishnan A., Verma V.K., Bronk S.F., Werneburg N.W., Charlton M.R., Shah V.H., Malhi H., Gores G.J. (2016). Lipid-Induced Signaling Causes Release of Inflammatory Extracellular Vesicles From Hepatocytes. Gastroenterology.

[51] Zhao H., Shang Q., Pan Z., Bai Y., Li Z., Zhang H., Zhang Q., Guo C., Zhang L., Wang Q. (2018). Exosomes From Adipose-Derived Stem Cells Attenuate Adipose Inflammation and Obesity Through Polarizing M2 Macrophages and Beiging in White Adipose Tissue. Diabetes.

[52] He X., Dong Z., Cao Y., Wang H., Liu S., Liao L., Jin Y., Yuan L., Li B. (2019). MSC-Derived Exosome Promotes M2 Polarization and Enhances Cutaneous Wound Healing. Stem Cells Int..

[53] Zhao J., Li X., Hu J., Chen F., Qiao S., Sun X., Gao L., Xie J., Xu B. (2019). Mesenchymal stromal cell-derived exosomes attenuate myocardial ischaemia-reperfusion injury through miR-182-regulated macrophage polarization. Cardiovasc. Res..

[54] Ti D., Hao H., Tong C., Liu J., Dong L., Zheng J., Zhao Y., Liu H., Fu X., Han W. (2015). LPS-preconditioned mesenchymal stromal cells modify macrophage polarization for resolution of chronic inflammation via exosome-shuttled let-7b. J. Transl. Med..

[55] Rosenberger C.M., Podyminogin R.L., Navarro G., Zhao G.W., Askovich P.S., Weiss M.J., Aderem A. (2012). miR-451 regulates dendritic cell cytokine responses to influenza infection. J. Immunol..

[56] Okamoto M., Fukushima Y., Kouwaki T., Daito T., Kohara M., Kida H., Oshiumi H. (2018). MicroRNA-451a in extracellular, blood-resident vesicles attenuates macrophage and dendritic cell responses to influenza whole-virus vaccine. J. Biol. Chem..

[57] Zhang F., Huang W., Sheng M., Liu T. (2015). MiR-451 inhibits cell growth and invasion by targeting CXCL16 and is associated with prognosis of osteosarcoma patients. Tumor Biol..

[58] Zhang J., Xu Z., Kong L., Gao H., Zhang Y., Zheng Y., Wan Y. (2020). miRNA-486-5p Promotes COPD Progression by Targeting HAT1 to Regulate the TLR4-Triggered Inflammatory Response of Alveolar Macrophages. Int. J. Chronic Obstr. Pulm. Dis..

[59] Jiang J., Gao Q., Gong Y., Huang L., Lin H., Zhou X., Liang X., Guo W. (2018). MiR-486 promotes proliferation and suppresses apoptosis in myeloid cells by targeting Cebpa in vitro. Cancer Med..

[60] Lai L., Song Y., Liu Y., Chen Q., Han Q., Chen W., Pan T., Zhang Y., Cao X., Wang Q. (2013). MicroRNA-92a negatively regulates Toll-like receptor (TLR)-triggered inflammatory response in macrophages by targeting MKK4 kinase. J. Biol. Chem..

[61] Dibble C.C., Cantley L.C. (2015). Regulation of mTORC1 by PI3K signaling. Trends Cell Biol..

[62] Weichhart T., Hengstschlager M., Linke M. (2015). Regulation of innate immune cell function by mTOR. Nat. Rev. Immunol..

[63] Rocher C., Singla D.K. (2013). SMAD-PI3K-Akt-mTOR pathway mediates BMP-7 polarization of monocytes into M2 macrophages. PLoS ONE.

[64] Reis P.P., Drigo S.A., Carvalho R.F., Lopez Lapa R.M., Felix T.F., Patel D., Cheng D., Pintilie M., Liu G., Tsao M.S. (2020). Circulating miR-16-5p, miR-92a-3p, and miR-451a in Plasma from Lung Cancer Patients: Potential Application in Early Detection and a Regulatory Role in Tumorigenesis Pathways. Cancers.

[65] Small E.M., O’Rourke J.R., Moresi V., Sutherland L.B., McAnally J., Gerard R.D., Richardson J.A., Olson E.N. (2010). Regulation of PI3-kinase/Akt signaling by muscle-enriched microRNA-486. Proc. Natl. Acad. Sci. USA.

[66] Ponnudurai T., Leeuwenberg A.D., Meuwissen J.H. (1981). Chloroquine sensitivity of isolates of Plasmodium falciparum adapted to in vitro culture. Trop. Geogr. Med..

[67] Karl S., Wong R.P., St Pierre T.G., Davis T.M. (2009). A comparative study of a flow-cytometry-based assessment of in vitro Plasmodium falciparum drug sensitivity. Malar. J..

[68] Mu J., Myers R.A., Jiang H., Liu S., Ricklefs S., Waisberg M., Chotivanich K., Wilairatana P., Krudsood S., White N.J. (2010). Plasmodium falciparum genome-wide scans for positive selection, recombination hot spots and resistance to antimalarial drugs. Nat. Genet..

[69] Rathod P.K., McErlean T., Lee P.C. (1997). Variations in frequencies of drug resistance in Plasmodium falciparum. Proc. Natl. Acad. Sci. USA.

[70] Mungchan P., Glab-Ampai K., Chruewkamlow N., Trakarnsanga K., Srisawat C., Nguyen K.T., Chaicumpa W., Punnakitikashem P. (2022). Targeted Nanoparticles for the Binding of Injured Vascular Endothelium after Percutaneous Coronary Intervention. Molecules.

[71] Xiao C.T., Lai W.J., Zhu W.A., Wang H. (2020). MicroRNA Derived from Circulating Exosomes as Noninvasive Biomarkers for Diagnosing Renal Cell Carcinoma. OncoTargets Ther..

[72] Lange T., Stracke S., Rettig R., Lendeckel U., Kuhn J., Schluter R., Rippe V., Endlich K., Endlich N. (2017). Identification of miR-16 as an endogenous reference gene for the normalization of urinary exosomal miRNA expression data from CKD patients. PLoS ONE.

[73] Wang X., Zhang X., Yuan J., Wu J., Deng X., Peng J., Wang S., Yang C., Ge J., Zou Y. (2018). Evaluation of the performance of serum miRNAs as normalizers in microRNA studies focused on cardiovascular disease. J. Thorac. Dis..

