# Supplementary material for: Differential Effect of Extracellular Vesicles Derived from Plasmodium falciparum-Infected Red Blood Cells on Monocyte Polarization
Source: Int J Mol Sci. 2023 Jan 30;24(3):2631. doi: 10.3390/ijms24032631 (PMC9916780; doi:10.3390/ijms24032631)
Supplement: Supplementary file 1 [file ijms-24-02631-s001.zip › ijms-2166326-supplementary.pdf]

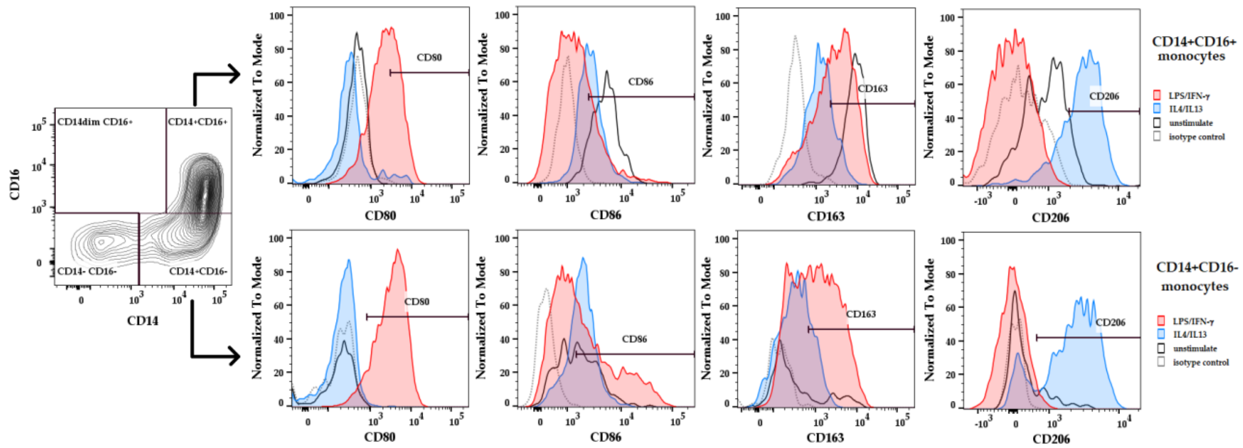

Figure S1 Representative flow cytometric analysis of cell surface markers (CD80, CD86, CD163, and CD206) expression on primary monocyte stimulated with LPS/IFN-γ (red histogram) and IL-4/IL-13 (blue histogram) compared to unstimulated cells (black-lined histogram). The upper panel showed the cell surface marker expression level of the $CD14^{+}CD16^{+}$ monocyte population, and the lower panel showed the expression level of the $CD14^{+}CD16^{-}$ monocyte population. The percentages of positive cells for each cell surface marker were identified based on fluorescent-labeled isotype-match antibodies (dot histogram).
